# ON THE RELATIONSHIP BETWEEN SUBJECTIVE AGE AND GRIP STRENGTH IN OLDER POPULATIONS

**DOI:** 10.1093/geroni/igae098.2348

**Published:** 2024-12-31

**Authors:** Omar Paccagnella

**Affiliations:** University of Padova, Padova, Veneto, Italy

## Abstract

Subjective age is the extent to which an individual feels younger or older than his/her chronological age. A large amount of literature shows that feeling younger is associated with better physical, psychological and cognitive outcomes. In this work, we exploit the richness and the comparability of three sister surveys on ageing (HRS, ELSA, SHARE, using both the Gateway to Global Aging Data platform and the original datasets) to investigate the relationship between subjective age and handgrip strength, that can be thought as an objective measure of physical health. Three different countries (UK, US, Israel) are separately analyzed and data on subjective age are collected in three different time periods (2004, 2008, 2015, respectively). The common framework is provided by the grip measurements that are collected four years later than subjective age answers in each survey. A set with the same harmonized characteristics across all countries is identified and used in the final estimations. The Heckman selection model is the adopted solution, to take sample selection bias (due to attrition) into account. Models are estimated by gender. The main results of our analysis are: i) controlling for selection bias, no statistically significant relationship between subjective age and grip strength appears in any country, reversing many findings we can observe when selectivity effects are neglected; ii) subjective age can be a promising exclusion variable when a selection approach is adopted (individuals who feel younger than their chronological age are more likely to collect data in subsequent survey waves).

